# Perceptions and recommendations by scientists for a potential release of genetically modified mosquitoes in Nigeria

**DOI:** 10.1186/1475-2875-13-154

**Published:** 2014-04-23

**Authors:** Patricia N Okorie, John M Marshall, Onoja M Akpa, Olusegun G Ademowo

**Affiliations:** 1 Institute for Advanced Medical Research and Training, College of Medicine, University of Ibadan, Ibadan, Nigeria; 2 Department of Infectious Disease Epidemiology, MRC Centre for Outbreak Analysis and Modelling, Imperial College London, London W2 1PG, UK; 3 Department of Epidemiology and Medical Statistics, College of Medicine, University of Ibadan, Ibadan, Nigeria

**Keywords:** Genetically modified mosquitoes, Malaria, Nigeria, Scientists, Perception of GMM, Biotechnology

## Abstract

**Background:**

The use of genetically modified mosquitoes (GMMs) for the control of malaria and other mosquito-borne diseases has been proposed in malaria-endemic countries, such as Nigeria, which has the largest burden in Africa. Scientists are major stakeholders whose opinions and perceptions can adversely affect the success of the trials of GMMs if they are not involved early. Unfortunately, information on the awareness of Nigerians scientists and their overall perception of the GMMs is practically non-existent in the literature. Therefore, this study aimed at understanding how receptive Nigerian scientists are to a potential release of GMMs for the control of malaria.

**Methods:**

The sample consisted of 164 scientists selected from academic and research institutions in Nigeria. Data were collected from participants using a semi-structured, self-administered questionnaire. Questions were asked about the cause and prevention of malaria, genetic modification and biotechnology. Specific questions on perception and acceptable conditions for the potential release of GM mosquitoes in Nigeria were also covered.

**Results:**

All participants cited mosquitoes as one of several causes of malaria and used various methods for household control of mosquitoes. The main concerns expressed by the scientists were that GMMs can spread in an uncontrolled way beyond their release sites (89%) and will mate with other mosquito species to produce hybrids with unknown consequences (94.5%). Most participants (92.7%) agreed that it was important that before approving the release of GMMs in Nigeria, there had to be evidence of contingency measures available to remove the GMMs should a hazard become evident during the course of the release. In general, a majority (83.5%) of scientists who participated in this study were sceptical about a potential release in Nigeria, while 16.5% of the participants were in support.

**Conclusions:**

Although a majority of the participants are sceptical about GMMs generally, most encourage the use of genetic modification techniques to make mosquitoes incapable of spreading diseases provided that there are contingency measures to remove GMMs if a hazard becomes evident during the course of the release.

## Background

Malaria is the world’s most important vector-borne disease and is widespread throughout the tropical and subtropical regions of the world [1]. It is caused by infection with a protozoan parasite of the genus *Plasmodium* and transmitted by *Anopheles* mosquitoes. In Nigeria, the main vectors are *Anopheles gambiae s.s., Anopheles arabiensis, Anopheles melas* and *Anopheles funestus*[2]. Nigeria has the largest burden of malaria in Africa with over 100 million people at risk of malaria every year [1,3]. Generally, the strategy of malaria control is based on chemotherapy and breaking the chain of transmission of the parasites between humans and mosquitoes [1]. Currently, malaria vector control mainly involves the use of insecticide-treated bed nets (ITNs) and/or long-lasting insecticidal nets (LLINs), and indoor residual spraying (IRS) [1]. However, malaria control programmes in Africa have not been very successful due to widespread insecticide resistance, limited resources and difficulties in implementing the available strategies on a large scale [4-6]. The failure of existing control measures has led to interest in the development of new approaches to reduce the burden of malaria. Several new approaches involve the use of genetically modified mosquitoes (GMMs), some of which are engineered to be genetically sterile and hence induce population suppression, while others have been engineered such that they are incapable of transmitting *Plasmodium*, with the goal being to spread this trait into a population [5,7-9].

The first open releases of GMMs took place in the Cayman Islands in 2009 and 2010 where three million genetically sterile *Aedes aegypti* mosquitoes, the main vector of dengue fever, were released with the intention of reducing their population size [4,5,7,8]. Subsequently, in 2009–2011, genetically sterile *Ae. aegypti* mosquitoes were released in large numbers in Brazil (ten million) as well as in Malaysia (6,000) [5,7]. The sterile GMMs were engineered to have a gene that causes 96% of offspring to die before reaching maturity [8]. The intention here is that as genetically sterile males mate with wild females, the reproductive potential of the females will be wasted, thereby reducing the mosquito population size. In these trials, released male GMMs were found to be half as successful in mating as wild ones and this rate was found to be sufficient to suppress the population [8]. Another genetically sterile strain of *Ae. aegypti* was evaluated for its potential to enhance dengue prevention efforts by inducing population suppression in a large field cage experiment in Mexico [10]. The results here showed that, although there was a significant decrease in the target population size, none of the treatment populations were eliminated, possibly due to a fitness disadvantage associated with the genetically modified strain [10].

The lessons that have been learnt from the GM crop controversy in Europe and North America would suggest that for GMMs to be acceptable for malaria control, scientists need to involve the public prior to trials, as well as during the research process and the development of such sophisticated tools [7]. There is much speculation surrounding genetic modification from pressure groups in various parts of the world and most misconceptions are a result of lack of accurate information. For example, Oxford Insect Technologies (Oxitec) faced a backlash from non-governmental organizations and the public in the case of release of sterile *Ae. aegypti* in the Cayman Islands [7]. Oxitec was criticized for their financial interest to public health by dealing with GMMs as a commercial product and releasing the GMMs before international regulations were set [7,11]. Oxitec was also accused of not publicly announcing the releases in the Cayman Islands and deliberately conducting the release in secret and then publishing the release after a one year delay [7,12]. GMMs generate debate in many parts of the world because of the prospect of releasing flying transgenic organisms into the environment. Before a field trial is conducted, it is imperative to find out the attitudes and concerns of people towards the potential release of GMMs. Through better communication and transparency prior to a release in Africa, such controversies like that generated by the release in Cayman Islands could be avoided.

Few surveys have been conducted to discover the views of people towards GMMs for disease control. In a survey carried out in 2003 and 2004 to gauge attitudes of the Japanese people to bioethics, a few questions were posed relating to genetically modified organisms (GMOs) [13]. These surveys focused specifically on whether the use of GMMs “unable to be a vector for human diseases like malaria or Japanese encephalopathy” was acceptable to the Japanese people. The most comprehensive study was a qualitative survey to determine public attitudes to GMMs for malaria control in Mali [14]. This survey focused on GMMs unable to transmit malaria and the “population replacement” strategy whereby malaria-refractory transgenes are linked to a gene drive system capable of manipulating inheritance in their favour [15]. The authors reported that participants wanted evidence that GMMs can reduce malaria prevalence without negative consequences to human health and the environment before they could agree to the release of GMMs. Recently, a questionnaire survey was conducted to determine how scientists working on malaria and its vector mosquitoes perceive public opinion and how they evaluate public consultations in their research [12]. The results from this study suggest that malaria researchers agree to interact with a non-scientific audience. Scientists are major stakeholders whose opinions and perceptions can adversely affect the success of trials of GMMs if they are not involved early [16].

The opinion of Nigerian scientists on GMMs is critical to potential trials, not only in the country itself but also in Africa at large, as Nigeria is the most populous country in the continent. Apart from providing explanations and information to the public, scientists are expected to provide guidance to decision-makers with regard to policies related to the potential release of GMMs for the control of malaria [9]. Unfortunately, information on the awareness of Nigerian scientists and their overall perception of GMMs is practically non-existent in the literature. This study aimed to understand how receptive Nigerian scientists are to the potential release of GMMs for the control of malaria. The survey addresses the use of GMMs unable to transmit malaria and the population replacement strategy whereby transgenes are driven into a population. Such a strategy is thought to be most appropriate for disease control on a wide scale [15], as it can be effective beyond the intervention site and over a long time scale. This is in contrast to sterile GMM releases, which are only effective at the site of intervention and over a short time period; however, ethical and regulatory issues must be confronted regarding the spread of transgenes between communities and across international borders [17].

## Methods

### Participants and sampling strategy

A total of 164 scientists were selected from different disciplines (science, medicine, agriculture). While participants were selected to obtain a mixture of backgrounds, only scientists working in academia, research institutes and tertiary health facilities were interviewed. In brief, two states in Nigeria (Oyo and Kwara state) were purposively selected from the southern and northern Nigeria respectively. In each state, institutions in the state capital with scientists working in areas related to Agriculture, Science and Health sciences were selected. As many scientists who gave informed consent were interviewed in the two states. A total of 157 scientist participated in the study in Oyo state while due to difficulties in obtaining consents, only seven participants were interviewed in Kwara state.

### Questionnaire, measures and data collection

A semi-structured questionnaire was designed and pre-tested among 60 respondents (scientists) selected from different institutions in Nigeria. The experiences of the pre-test were used to refine the final questionnaire used for the main study. The questionnaire related to general information on malaria, genetic modification and biotechnology and included questions on the demographic characteristics of the participants. Regarding GMMs, participants were asked to imagine that an organization from a foreign country claimed they could provide a GMM capable of reducing malaria morbidity and mortality through population replacement with malaria-refractory mosquitoes. In addition, they were told that, to the best of their knowledge, the GMMs were safe, although there could be unknown negative consequences. Questions were then asked assessing participants’ conditions for approving a release of GMMs in Nigeria, their specific concerns and general issues about GMM releases in Nigeria. Scientists’ perceptions were measured using a five-point Likert scale instrument containing ten items assessing participants’ perceptions about GMMs (both negative and positive) developed for this study. The ten items were gathered from the literature [7,9,12,14,18,19] and relevant local issues that were considered crucial to the objectives of this study. The five points were coded as follows: 1: strongly disagree (SD); 2: disagree (D); 3: neutral (N); 4: agree (A); and, 5: strongly agree (SA). Data were collected from participants using the semi-structured, self-administered questionnaire, while trained interviewers were available in case participants required clarification. Each participant was interviewed independently in a secluded atmosphere by trained interviewers who were instructed not to provide assistance that could bias or influence participants’ responses, and only to help participants understand the questionnaire should the need arise.

### Data management and analysis

Data from collated questionnaires were coded and entered into the computer system after respondents’ identities were removed. Data were initially entered into an Excel spreadsheet and checked for outliers, entry errors and omissions. The cleaned data were then imported into Statistical Package for Social Sciences (SPSS® version 15) (SPSS) where further data exploration and cleaning was performed prior to statistical analysis. Scores for negatively worded perception questions in the perception scale were recorded in opposite direction to reflect positive perceptions. Perception total scores (PTS) for a participant were computed as the sum of scores a participant gave to each question in the ten-item scale (hence minimum and maximum total score for an individual was ten and 50, respectively). Mean ($\bar{X}$) and standard deviation (*SD*) of PTS were computed and $\mathrm{PTS}<\bar{X}+\mathrm{SD}$ were coded 1 (Sceptical perception) while $\mathrm{PTS}\geq \bar{X}+\mathrm{SD}$ were coded 2 (and considered positive/supportive perception). Frequency tables, percentages, means, and standard deviations were used as descriptive statistics for preliminary data analysis and explorations [20]. The Chi-squared test was used to screen for the dependence of GMM perception on selected variables (sex, age, educational level and the all other variables listed in Additional file 1). In an adjusted analysis, logistic regression was used to further assess the variables (number of children, usefulness and risk of GMMs) found from the chi-square test to significantly associate with GMM perception among participants (see Additional file 1). All analyses were performed at 95% confidence level using SPSS.

### Ethical clearance

Ethical approval for this study was obtained from the University of Ibadan and the University College Hospital, Ibadan (UI/UCH) Ethics Review Committee with approval number UI/UCH/EC/12/0247. Furthermore, in line with the guidelines of the institution review committee, the following ethical issues were addressed: (1) Confidentiality of data: confidentiality was protected by using identifying code numbers for each participant. The survey was anonymous. (2) Beneficence to participants: The participants’ interest in GMMs were awakened and they were able to share their views on GMM. (3) Non-maleficience to the participants: Precautions were taken to reduce to the barest minimum any form of inconvenience to the participants during the study. (4) Justice: Method of participants selection was scientifically objective and fairness was assured.

## Results

### Demography

A total of 164 scientists completed the self-administered questionnaire. A majority of the scientists were males (61.6%) while 36.0% were female (Table 1). A majority (74.4%) were aged between 30 and 49 years, while 20.7% were over 49 years. More than three-quarters (86.6%) had completed advance degrees (MSc: 45.7%; PhD: 40.9%). Most of those who participated in the study (59.2%) were scientists with long-standing academic/research experiences (Table 1).

**Table 1:** Sociodemographic characteristics of respondents

**Variables**	**Frequency**	**Percentage (N = 164)**
*Age*		
<30	8	4.9
30-49	122	74.4
>49	34	20.7
*Gender*		
Male	101	61.6
Female	59	36.0
Not reported	4	2.4
*Number of children*		
None	17	10.4
1-3	103	62.8
4 and above	35	21.3
Not specified	9	5.5
*Highest formal educational qualification*		
First degree/Higher diploma	20	12.2
Masters degree	75	45.7
Doctoral degree	67	40.9
Not reported	2	1.2
*Years of experience in academia/research*		
5 or less	65	39.6
6-15	70	42.7
>15	27	16.5
Not reported	2	1.2

### Causes of malaria and malaria protection method

Most of the scientists (97.6%) attributed the cause of malaria to mosquito bites (see Additional file 2) while 41.4% and 60.4%, respectively considered stress/overwork and presence of stagnant water as possible causes of malaria. Of the four respondents that did not include mosquito bites as the main cause of malaria, none encouraged the use of GMMs for disease control, two (50%) felt it will never be feasible to release GMMs in Nigeria, while two (50%) were not sure. Their major concerns about GMMs included the possibilities of GMM transmitting other diseases, spreading uncontrollably beyond the release sites, or becoming resistant to insecticides and fogging. Screening of windows/doors (89.6%), use of aerosols (86.6%), use of long-lasting insecticidal nets (LLINs) (84.8%) and environmental management (66.5%) were the most frequently mentioned malaria preventive methods by the scientists (see Additional file 2).

### Knowledge and perception of genetic modification and applications of biotechnology

Almost one-third (31.7%) of the scientists had heard or read a great deal about genetic modification while 60.4% of them had heard or read about genetic modification somewhat (see Additional file 2). Some of the participants (40.2%) had heard about the application of biotechnology to make mosquitoes unable to transmit diseases; more than half (56.1%) agreed that mosquitoes unable to transmit diseases would be useful to society, but 17.7% believed that such technology is risky (see Additional file 2).

### Source of information on genetic modification

**Figure 1:**
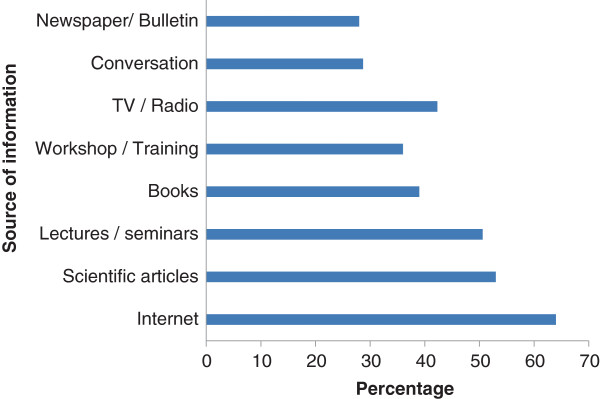
Scientists’ sources of information on genetic modification.

Figure 1 shows that Nigerian scientists gained information about GMMs from a variety of sources. The internet (accounting for 64.3%) was the most popular source of information while others sourced information on GMMs from newspapers/bulletins (27.4%) and from conversations (29.3%) with other scientists.

### Scientists’ concerns and recommendations for releasing GMMs in Nigeria

The main concerns expressed by the scientists were that GMMs can spread in an uncontrolled way beyond the release sites and that GMMs will mate with other mosquito species to produce hybrids with unknown consequences. Over 68% of the participants indicated that they were very concerned with both issues (Table 2). Other major concerns were that GMMs could transmit other unknown diseases and may also become resistant to insecticides and fogging (Table 2). A prominent concern (expressed by 47.6%) was that GMMs would continue to transmit malaria.

**Table 2:** Scientists’ concerns for releasing genetically modified mosquitoes in Nigeria

**Scientists’ concerns**	**% of responses**			
**Concerns about GMM**	**Very concerned**	**A little concerned**	**Not concerned**	**Not sure**
1. GM mosquitoes can spread in an uncontrolled way beyond their release sites	68.9	20.1	9.8	1.2
2. GM mosquitoes will mate with other mosquito species producing hybrids with unknown consequences	68.3	26.2	4.3	1.2
3. GM mosquitoes will transmit other unknown diseases	65.2	28.2	6.1	0.6
4. GM mosquitoes will become resistant to insecticides and fogging	64.6	28.2	6.7	0.6
5. GM mosquitoes will harm the ecosystem	60.4	29.9	9.1	0.6
6. GM mosquitoes will be too expensive for developing countries	50.0	33.5	14.6	1.2
7. GM mosquitoes will continue to transmit malaria	47.6	37.2	12.8	1.8

Before deciding whether they approved of GMMs being released in Nigeria, scientists were asked to rate a set of recommendations/requirements in order of importance to them (ranging from very important to not important). A majority (81.5%) felt that “evidence of contingency measures available to remove GMMs if a hazard becomes evident during the course of the release” was very important (Table 3). However, 15.2% felt provision of bed nets as a requirement for releasing GMMs in Nigeria was not important.

**Table 3:** Scientists’ recommendations for releasing genetically modified mosquitoes in Nigeria

**Scientists’ recommendations/requirements**	**Very important**	**Moderately important**	**Not important**	**Not sure**
1. Evidence of contingency measures available to remove the GM mosquitoes if a hazard becomes evident during the course of release	81.7	11.0	1.8	2.4
2. Education campaigns on how GM mosquitoes reduce malaria	78.0	13.4	4.3	2.4
3. A confirmed trial in a community in Nigeria	76.8	10.4	5.5	3.0
4. Scientific evidence that it is possible to reduce malaria using GM mosquitoes	70.7	20.1	4.3	3.0
5. Mosquitoes to be modified captured from our environment	65.2	25.6	4.3	3.7
6. Approval from Nigerian government	65.9	24.4	5.5	1.8
7. Dialogue between Nigerian government, general public and scientists who modify mosquitoes	62.2	22.6	7.3	5.5
8. Evidence from a trial in another African country	62.2	22.6	7.3	5.5
9. Approval from a majority of the Nigerian public	55.5	26.8	15.2	1.2
10. Bed nets provided	51.8	25.0	15.2	5.5

The most trusted organizations on safety assessment of GMMs among the scientists were World Health Organization (WHO) (82.3%) and universities/research institutes (56.1%) (Table 4). The Nigerian government was the least trusted (11.0%), with almost half of participants having no trust at all in the government for a safety assessment of GMMs release in Nigeria (Table 4).

**Table 4:** Trusted organizations for safety assessment of genetically modified mosquitoes

**Organization**	**Trust a lot**	**Trust moderately**	**Do not trust**	**Not sure**	**No response**
WHO	82.3	11.6	1.8	1.2	3.0
Universities/research institutes	56.1	37.8	1.8	1.2	3.0
International media	30.5	37.2	18.3	3.7	10.4
Foreign biotechnology companies	35.4	33.5	20.1	3.7	7.3
Religious bodies	27.4	30.5	16.5	12.2	13.4
Nigerian media	16.5	36	29.9	8.5	9.1
Nigerian government	11	28	49.4	3.7	9.1

### Participants’ perception of GMMs

#### Negative statements

About 33.9% of the participants agreed that GMMs would have little effect on malaria, as has been the case for bed nets and insecticides, which have only slightly reduced the number of malaria cases in Africa. Some 15.8% agreed that GMMs should not be released as they would have unknown risks. A very few (12.2%) considered it likely that there will be horizontal gene transfer from GMMs to humans, with unknown consequences, and fewer (7.3%) considered it likely that GMMs may produce new metabolites or toxins likely to have deleterious effects on parasites or predators (see Additional file 3).

#### Positive statements

Some 76.2% agreed to approve the release of GMMs but only if they saw the results of successful laboratory experiments, while 73.2% agreed that it is better to modify mosquitoes so they cannot transmit diseases since other methods that have attempted to eradicate mosquitoes have not worked (see Additional file 3). About 56.1% strongly agreed that GMMs would improve the quality of living in developing countries and almost half (48.2%) agreed that malaria is far worse than any negative consequences that the GMMs might have. In general, a majority (83.5%) who participated in this study were sceptical about a potential release of GMMs in Nigeria, while 16.5% were in support (see Additional file 2).

#### Relationship between perception and other factors

Results of the Chi-squared test (see Additional file 1) shows how the number of children that participants have significantly correlates with their perception of GMMs (*χ*^2^ = 7.80, p < 0.05). Among participants who were sceptical about a potential release of GMMs in Nigeria, the proportion of those with more than four children (88.6%) was significantly higher than the proportion of those with no children (58.8%). Only 21.3% of participants had more than four children. Similarly, perception of GMMs was significantly correlated with how participants considered the usefulness of GMMs (*χ*^2^ = 6.40, p < 0.05). Among participants who were sceptical about a release of GMMs in Nigeria, the proportion of those who consider GMMs are not useful (94.4%) was significantly higher than the proportion of those who consider GMMs useful (77.2%). The adjusted logistic analysis (at 5% significance level) shows that perception about GMM was not associated with any factor in this study.

#### General questions on genetically modified mosquitoes

In general, 66.5% of the scientists agreed that the use of genetic modification techniques to make mosquitoes incapable of spreading diseases, such as malaria, should be encouraged while 15.9% opposed such techniques. Preference for who should be involved in the modification, 33.9% of the participants preferred local scientists, 37.3% preferred international scientists, while 25.8% preferred both local and international scientists to be involved.

About 56% of the scientists think use of GMMs that are unable to transmit diseases is valuable for the society while 17.7% think it is risky (see Additional file 2). Generally, about two-thirds (66.5%) of the scientists agreed that the use of genetic modification techniques to make mosquitoes incapable of spreading diseases, such as malaria, should be encouraged. More than half (58.5%) interviewed thought that it will be feasible to release GMMs in Nigeria.

## Discussion

Public attitudes to disease control strategies using GMMs are particularly important given the controversy that has followed trials of GMMs in several countries [7], and in the light of potentially dangerous health consequences of modifying a disease vector. It is necessary to gauge whether scientists are open to the potential release of GMMs and if the technology is acceptable to them. Indeed, it is essential to engage the community in early stages of research when new technologies are being developed and tested so as to get the communities to accept it [9]. Effort was made in the present study to obtain the overall considerations of Nigerian scientists on a potential release of GMMs in Nigeria. Although public surveys were very well received, one must be careful when interpreting these results. For instance, this survey was far from a random sample as the selection of participants was mainly purposive. Consequently, despite the rigorous efforts to engage only those participants who could provide appropriate information in the study, the results presented here are considered a subset of the opinion of scientists in Nigeria and their representativeness is debatable. Hence, it is impossible to generalize the findings of the present study to the entire Nigerian scientists. Non-response bias could be a factor here as some that did not respond may be more or less supportive of GMM technology. Nevertheless, they do provide a range of perspectives of scientists across the country. The current findings can inform not only development of subsequent surveys but also policy decisions regarding a potential release of GMMs.

As expected, almost all scientists agreed that malaria is caused by bites from infected mosquitoes. This awareness is high and can likely be attributed to the level of education and exposure of the respondents. It also suggests that an adequate sample has been taken for the present study as most of the participants (the scientists) will have meaningful opinions about mosquito control methods, and may have thought of alternative mosquito control methods. Understandably, almost all the respondents perceived mosquitoes to be the main cause of malaria. It was found that the majority of participants had heard or read something about genetic modification with some confirming that they had heard or read a great deal about it. Although the information about their specific motivations is lacking, it is not surprising to have scientists updating their knowledge on emerging technologies most especially when it is in the premise of their research background or interests. On the other hand, less than half of the participants had heard about the application of biotechnology to make mosquitoes unable to transmit diseases. This is understandable since no trial of GMMs has been conducted in Nigeria, although given the background of these scientists; it suggests that there is low awareness of GMMs amongst them.

The majority of the participants believed that GMMs could be useful for malaria control and that the risks involved are minimal. Proponents of malaria-refractory GMMs have proposed that they have the advantage of being species-specific, they could reduce the need for the use of insecticides, they would protect everyone in the release area, irrespective of socio-economic class or status, and would require less community involvement in the control method [4-6,9]. The main concerns of Nigerian scientists about malaria-refractory GMMs are that they may spread in an uncontrolled way beyond the release sites, which may result in hybrid mosquitoes having unknown consequences. Other major concerns included the fear that GMMs will transmit unknown diseases and may become resistant to insecticides and fogging. A less commonly expressed concern was that GMMs would continue to transmit malaria. The modification of mosquitoes such that they are incapable of spreading disease is risky in the context of biological adaptability and resistance as little is known about the behaviour of GMMs in the field [7]. It is not known how GMMs will respond in terms of behaviour, biological fitness and how transgenic mosquitoes will ultimately impact insect ecology. The use of genetically modified technology is controversial and some organizations fear that reliance on such solutions detracts from more effective but poorly deployed measures to control disease vectors [7]. These organizations fear that genetically modified insects could have unintended and wide-ranging impact on the environment and human health as they may lead to new public health problems by filling an ecological niche left by the wild insects that have been suppressed or eliminated [6]. In particular, there could be horizontal gene transfer and it may be impossible to reverse any damage caused by the introduced genetically modified insects. The general belief among most communities is that genetic modification is unnatural and thus undesirable and this is understandable as use of GMMs for malaria control involves the release of large numbers of sterile mosquitoes [6,9].

The most important or popular recommendation/requirement for scientists to approve the release of GMMs in Nigeria was that there had to be evidence of contingency measures available to remove GMMs if a hazard becomes evident during the course of the release. This implies that Nigerian scientists may be willing to support the use of GMMs provided a plan is available that could halt its use if something went wrong. Education campaigns on GMMs, a confirmed trial in a community in Nigeria and scientific evidence that GMMs can reduce malaria were also important requirements for the scientists. These requirements are not surprising since the participants (consisting of people from academia and research institutes) will most likely require scientific backing for their actions. Failure to adhere to recommendation by experts could have negative consequences on the outcome of some medical and technology trials [7,19].

Most laboratories involved in GMM development are located in western countries and not in countries where malaria is endemic and where GMMs may eventually be released in the field [7]. As this survey has shown, local scientists prefer to be involved in the development process and this can remove the need for transfer from laboratories in the West to the field. This is particularly encouraging as there has been a call for more scientists from disease-endemic countries to become involved in various aspects relating to the use of GMMs for disease control [9]. There is a need for partnership between scientists in developed and disease-endemic countries [16]. Further, the results of this survey suggest that, despite scientists in research and academia being knowledgeable, this does not translate to unconditional support of GMMs. This is in agreement with the findings in a study in Mali where Western-trained doctors and scientists were not more supportive of GMMs or biotechnology than those in the rural areas with less education [14]. Slightly more than half of the respondents thought that it would ever be feasible to release GMMs in Nigeria and, although this is far from a consensus, it is a majority of those interviewed, which is promising for the technology. However, more than 80% of participants were sceptical about GMMs, which indicates that there is strong scepticism over acceptance and tolerance of new technology [16].

## Conclusions

Although the majority of participants were sceptical about GMMs generally, most of them encouraged their use provided that evidence of contingency measures are available to remove them should a hazard becomes evident during the course of the release. While the scenario discussed here is hypothetical, the results demonstrate the hypothesis set out to test. Nonetheless, there are limitations to be drawn from public attitudes to a project that has not yet been carried out as people may respond differently in the face of reality [9].

## Competing interests

The authors declare that they have no competing interests.

## Authors’ contributions

PNO and JMM conceived and designed the study. PNO performed the public attitude surveys. PNO and OMA analysed the survey data and wrote the manuscript. OGA and JMM gave conceptual advice and provided editorial feedback on the manuscript. All authors read and approved the final version of the manuscript.

## Supplementary Material

Additional file 1 Assessing factors associated with the scientists’ perception of genetically modified mosquitoes. Click here for file

Additional file 2 Respondents’ knowledge and other considerations about malaria and genetically modified mosquitoes. Click here for file

Additional file 3 Scientists’ perception of genetically modified mosquitoes. Click here for file
