# Supplementary material for: Perceptions and recommendations by scientists for a potential release of genetically modified mosquitoes in Nigeria
Source: Malar J. 2014 Apr 23;13:154. doi: 10.1186/1475-2875-13-154 (PMC4021343; doi:10.1186/1475-2875-13-154)
Supplement: Additional file 1 — Assessing factors associated with the scientists’ perception of genetically modified mosquitoes. [file 1475-2875-13-154-S1.docx]

**Additional file 1: Assessing factors associated with the scientists’ perception of genetically modified mosquitoes**

|  | Perception of GMM | | | Odds of being sceptical about GMM |
| --- | --- | --- | --- | --- |
| VARIABLE | Sceptical $n(\%)$  | Supportive $n(\%)$  | $\chi^{2}$ | OR (95% CI) |
| Sex | | | | |
| - Male | 81(81.2) | 19(18.8) | 0.73 |  |
| - Female | 51(86.4) | 8(13.6) |  |  |
| Age of participants | | | | |
| - <30 | 7(87.5) | 1(12.5) | 2.04 |  |
| - 30-49 | 99(81.1) | 23(18.9) |  |  |
| - >40 | 31(91.2) | 3(8.8) |  |  |
| Highest educational qualification | | | | |
| - First degree/higher diploma | 16(80.0) | 4(20.0) | 2.20 |  |
| - Masters degree | 66(88.0) | 9(12.0) |  |  |
| - Doctoral degree | 53(79.1) | 14(20.9) |  |  |
| Number of children | | | | |
| - None | 10(58.8) | 7(41.2) | 7.80* | 4.12(0.74-23.03) |
| - 1-3 | 87(84.5) | 16(15.5) |  | 1.32(0.33-5.39) |
| - $\geq4$ | 31(88.6) | 41(11.4) |  | - |
| Years of academic/research experience | | | | |
| - $\leq5$ | 55(84.6) | 10(15.4) | 0.33 |  |
| - 6-15 | 57(81.4) | 13(18.6) |  |  |
| - >15 | 23(85.2) | 4(14.8) |  |  |
| Have heard about genetic modification | | | | |
| - Not at all | 10(83.3) | 2(16.7) | 1.45 |  |
| - Somewhat | 80(80.8) | 19(19.2) |  |  |
| - A great deal | 46(88.5) | 6(11.5) |  |  |
| Have heard of mosquitoes unable to transmit diseases | | | | |
| - Yes | 56(84.8) | 10(15.2) | 1.91 |  |
| - No | 61(83.6) | 12(16.4) |  |  |
| - Not sure | 9(69.2) | 4(30.8) |  |  |
| Thinks mosquitoes unable to transmit diseases is useful | | | | |
| - Yes | 71(77.2) | 21(22.8) | 6.40* | 3.98(0.31-51.52) |
| - No | 17(94.4) | 1(5.6) |  | 1.71(0.06-47.14) |
| - Not sure | 22(95.7) | 1(4.3) |  | - |
| Thinks mosquitoes unable to transmit diseases is risky | | | | |
| - Yes | 27(93.1) | 2(6.9) | 10.06** | 0.61(0.07-5.47) |
| - No | 43(70.5) | 18(29.5) |  | 2.36(0.44-12.59) |
| - Not sure | 33(91.7) | 3(8.3) |  | - |
