# Supplementary material for: Perceptions and recommendations by scientists for a potential release of genetically modified mosquitoes in Nigeria
Source: Malar J. 2014 Apr 23;13:154. doi: 10.1186/1475-2875-13-154 (PMC4021343; doi:10.1186/1475-2875-13-154)
Supplement: Additional file 2 — Respondents’ knowledge and other considerations about malaria and genetically modified mosquitoes. [file 1475-2875-13-154-S2.docx]

**Additional File 2: Respondents’ knowledge and other considerations about malaria and genetically modified mosquitoes**

| Variable | Frequency | Percentage |
| --- | --- | --- |
| What do you think causes malaria? | | |
| Stress/overwork | 68 | 41.5 |
| Mosquito bites | 160 | 97.6 |
| Presence of stagnant water | 99 | 60.4 |
| How do you prevent malaria in your community? | | |
| Window/door nets | 147 | 89.6 |
| Use of aerosols | 142 | 86.6 |
| Insecticide-treated nets | 139 | 84.8 |
| Environmental management | 109 | 66.5 |
| Mosquito coil | 75 | 45.7 |
| Topical repellants | 56 | 34.1 |
| Protective clothing | 45 | 27.4 |
| Prophylaxis | 42 | 25.6 |
| Nothing at all | 12 | 7.3 |
| How much have you heard or read about genetic modification? | | |
| Nothing at all | 12 | 7.3 |
| Somewhat | 99 | 60.4 |
| A great deal | 52 | 31.7 |
| No response | 1 | 0.6 |
| Have you heard about mosquitoes unable to transmit diseases? | | |
| Yes | 66 | 40.2 |
| No | 73 | 44.5 |
| Not sure | 13 | 7.9 |
| No response | 12 | 7.3 |
| Do you think use of GMMs unable to transmit diseases is useful for the society? | | |
| Yes | 92 | 56.1 |
| No | 18 | 11.0 |
| Not sure | 23 | 14.0 |
| No response | 31 | 18.9 |
| Do you think the use of GMMs unable to transmit diseases is risky for society? | | |
| Risky | 29 | 17.7 |
| Not risky | 61 | 37.2 |
| Not sure | 36 | 22.0 |
| No response | 38 | 23.2 |
| Should the use of GMMs be encouraged? | | |
| Yes | 109 | 66.5 |
| No | 26 | 15.9 |
| Not sure | 27 | 16.5 |
| Not reported | 2 | 1.2 |
| Do you think it will ever be feasible to release GMMs in Nigeria? | | |
| Yes | 96 | 58.5 |
| No | 21 | 12.8 |
| Not sure | 45 | 27.4 |
| Not reported | 2 | 1.2 |
| Who should modify the mosquitoes? | | |
| Local scientists | 113 | 33.9 |
| International scientists | 124 | 37.3 |
| Both local and international scientists | 86 | 25.8 |
| Not sure | 10 | 3.0 |
| After this survey, will you seek more information on GMMs? | | |
| Yes | 135 | 82.3 |
| No | 18 | 11.0 |
| Not sure | 10 | 6.1 |
| Not reported | 1 | 0.6 |
| Overall perception of GMMs among scientists | | |
| Sceptical | 137 | 83.5 |
| Supportive | 27 | 16.5 |
