# Supplementary material for: Perceptions and recommendations by scientists for a potential release of genetically modified mosquitoes in Nigeria
Source: Malar J. 2014 Apr 23;13:154. doi: 10.1186/1475-2875-13-154 (PMC4021343; doi:10.1186/1475-2875-13-154)
Supplement: Additional file 3 — Scientists’ perception of genetically modified mosquitoes. [file 1475-2875-13-154-S3.docx]

**Additional file 3: Scientists’ perception of genetically modified mosquitoes**

| Perception item | **% of respondents** | | | | | |
| --- | --- | --- | --- | --- | --- | --- |
|  | Strongly disagree | Disagree | Neutral | Agree | Strongly agree | No response |
| 1. Malaria is far worse than any negative consequences that the GM mosquitoes could have | 14.0 | 16.5 | 21.3 | 25.6 | 22.6 | 0 |
| 1. If GM mosquitoes have unknown risks, they should not be released | 41.5 | 31.7 | 11.0 | 14.0 | 1.8 | 0 |
| 1. Bed nets and insecticides have barely reduced the number of malaria cases in Africa. GM mosquitoes will not be any different | 15.9 | 18.3 | 22.0 | 32.3 | 11.6 | 0 |
| 1. Scientists have tried to eradicate mosquitoes and it has not worked. It is better to modify them so they cannot transmit diseases | 5.5 | 9.1 | 11.6 | 40.9 | 32.3 | 0.6 |
| 1. If WHO tells me that GM mosquitoes will help reduce malaria morbidity and mortality, I will believe them | 35.4 | 47 | 11.6 | 3.7 | 1.2 | 1.2 |
| 1. There will be horizontal gene transfer from GM mosquitoes to humans with unknown negative consequences | 17.1 | 28.0 | 39.6 | 8.5 | 3.7 | 3.0 |
| 1. If I saw the results of a successful laboratory experiment, I will approve of release of GM mosquitoes | 3.7 | 7.9 | 12.2 | 37.8 | 38.4 | 0 |
| 1. GM mosquitoes will improve the quality of living in developing countries | 7.3 | 12.2 | 22.6 | 33.5 | 22.6 | 1.8 |
| 1. GM mosquitoes may produce 'new' metabolites or toxins likely to have deleterious effects on parasites or predators | 26.2 | 31.7 | 32.9 | 6.7 | 0.6 | 1.8 |
| 1. Humans are just playing God and interfering with nature | 21.3 | 15.9 | 23.2 | 18.9 | 18.9 | 1.8 |
